# VISTA/CTLA4/PD1 coexpression on tumor cells confers a favorable immune microenvironment and better prognosis in high-grade serous ovarian carcinoma

**DOI:** 10.3389/fonc.2024.1352053

**Published:** 2024-04-03

**Authors:** Aida Jlassi, Rim Rejaibi, Maroua Manai, Ghada Sahraoui, Fatma Zahra Guerfali, Lamia Charfi, Amel Mezlini, Mohamed Manai, Karima Mrad, Raoudha Doghri

**Affiliations:** ^1^ Research Laboratory of Precision Medicine/Personalized Medicine and Oncology Investigation Salah Azaiz Institute, Tunis, Tunisia; ^2^ Department of Biology, Mycology, Pathologies and Biomarkers Laboratory, Faculty of Sciences of Tunis, University of Tunis El Manar, Ariana, Tunisia; ^3^ Laboratory of Transmission, Control and Immunobiology of Infections, Pasteur Institute of Tunis, University of Tunis, El Manar, Tunis, Tunisia; ^4^ Department of Pathology, Salah Azaiez Institute, Tunis, Tunisia; ^5^ Medical Oncology Department, Salah Azaiez Institute, Tunis, Tunisia

**Keywords:** ovarian cancer, HGSOC, VISTA, CTLA4, PDL1, PD1, TILs, immunotherapy

## Abstract

**Introduction:**

Immunotherapy by blocking immune checkpoints programmed death/ligand (PD1/PDL1) and cytotoxic T-lymphocyte-associated protein 4(CTLA4) has emerged as new therapeutic targets in cancer. However, their efficacy has been limited due to resistance. A new- checkpoint V-domain Ig-containing suppressor of T cell activation (VISTA) has appeared, but the use of its inhibition effect in combination with antibodies targeting PDL1/PD1and CTLA4 has not been reported in ovarian cancer.

**Methods:**

In this study, we investigated the expressions of VISTA, CTLA4, and PDL1 using immunohistochemistry (IHC)on 135 Formalin-Fixed Paraffin-Embedded (FFPE)tissue samples of High-grade serous carcinoma (HGSOC). VISTA, CTLA4, PDL1, PD1, CD8, CD4, and FOXP3 mRNA extracted from 429 patients with ovarian cancer in the Cancer Genome Atlas (TCGA) database was included as a validation cohort. Correlations between these checkpoints, tumor-infiltrating- lymphocytes (TILs), and survival were analyzed.

**Results and discussion:**

CTLA4 was detectable in 87.3% of samples, VISTA in 64.7%, PD1 in 56.7%, and PDL1 in 48.1%. PDL1 was the only tested protein associated with an advanced stage (*p*=0.05). VISTA was associated with PDL1, PD1, and CTLA4 expressions (*p*=0.005, *p*=0.001, *p*=0.008, respectively), consistent with mRNA level analysis from the TCGA database. Univariate analyses showed only VISTA expression (*p*=0.04) correlated with overall survival (OS). Multivariate analyses showed that VISTA expression (*p*=0.01) and the coexpression of VISTA^+^/CTLA4^+^/PD1^+^ (*p*=0.05) were associated with better OS independently of the clinicopathological features. Kaplan-Meier analysis showed that the coexpression of the VISTA^+^/CTLA4^+^/PDL1^+^ and VISTA^+^/CTLA4^+^/PD1^+^ checkpoints on tumor cells (TCs)were associated with OS (p=0.02 and *p*<0.001; respectively). VISTA^+^/CTLA4^+^/PD1^+^ in TCs and CD4^+^/CD8^+^TILswere associated with better 2-yer OS. This correlation may refer to the role of VISTA as a receptor in the TCs and not in the immune cells. Thus, targeting combination therapy blocking VISTA, CTLA4, and PD1 could be a novel and attractive strategy for HGSOC treatment, considering the ambivalent role of VISTA in the HGSOC tumor cells.

## Introduction

The tumor microenvironment (TME) is enriched by the immune inflammatory cell, which plays crucial roles in tumor development, growth, progression, and therapy resistance (1). Immunotherapy is a promising new axis of cancer treatment due to its significant long-term clinical results and could improve the management of gynecological cancers, particularly epithelial ovarian cancer (EOC). It has been demonstrated that within the TME, co-inhibitory immune checkpoints can inactivate TILs. The literature shows that immunotherapy targeting the first generation of immune checkpoint molecules like CTLA-4 and PD-1 has proven effective in many cancers (2).In 2018, the Nobel Prize in Physiology and Medicine was awarded to two immunology researchers for discovering immunotherapy against lung cancer and melanoma by blocking CTLA4 and PD1 simultaneously (by anti-CTLA4 and anti-PD1 or anti-PDL1 monoclonal antibodies). However, not all patients responded to immune checkpoint inhibitors with a response rate of 30% after treatment.

EOC is an immunogenic tumor characterized by a high level of TILs, which improves the prognosis, but this is not always the case because of the immune escape mechanism of cancer cells from these TILs (1). VISTA as a regulator of antitumor immunity (3) by inhibiting T lymphocytes (4) was found to be highly expressed in several cancers such as ovarian cancer, endometrial cancer, gestational trophoblastic neoplasia and triple-negative breast cancer (TNBC) (3, 5, 6). Moreover, in the literature, VISTA expression was associated with metastases in ovarian cancer (7) and found to be frequently expressed in PDL1-negative HGSOC specimens (8). Additionally, previous *in vivo* experiments demonstrated that the combination therapy by blockading PD-L1 and VISTA synergistically affected proliferation and tumor growth in colon cancer models (9).

For certain advanced cancers, immune checkpoint blockers (ICBs) were approved by the Food and Drug Administration (FDA) to be used alone in front-line therapies or in combination with other regimens. The first generation of PD-1/PD-L1 and CTLA-4 immune checkpoint inhibitors was only sensitive in a subset of patients and has limited efficacy in treating ovarian cancer. The phenomena involved in resistance to immunotherapy are little known. The relationship between VISTA, PDL1, PD1, CTLA4, TILs, and prognosis in EOC remains unknown. In the present study, the objectives were to (i) characterize VISTA, PDL1, PD1, and CTLA4 expression in a large cohort of HGSOC using immunohistochemistry (IHC); (ii) to evaluate the correlation of VISTA, PD-L1, PD1, and CTLA4 with the clinicopathological characteristics, and the density of TILs; (iii) to evaluate the prognostic value of PD1/PDL1, CTLA4, and VISTA coexpression in terms of OS; (iv) and finally to validate our results on the mRNA expression level by using the TCGA database.

## Materials and methods

### Patient cohort and tissue microarray

In this study,135 HGSOC cases were collected and diagnosed at Salah Azaiez Institute (SAI) between 2000 and 2017. Approval by the institutional ethics committee of SAI was achieved.

The inclusion criteria included pathologically confirmed HGSOC, availability of pre-therapeutic diagnostic FFPE tumor samples, clinicopathological annotations including treatment and follow-up, and patient’s written informed consent. Patients who had incomplete medical records or adequate tumor and stromal contents for TMA cores were excluded. All tumor tissues were obtained at the time of primary surgery from patients who were not treated or subsequently received standard neoadjuvant chemotherapy. Consistent with our previous study, all cases were FFPE. All samples were spotted into one TMA as previously described (10, 11). Briefly, samples for TMA were collected using 1 mm diameter core needles from a spot of tumors with the most representative histology of each surgical specimen. For each sample, two representative areas were carefully selected. The TMA was embedded in the recipient paraffin block using a specific arraying device (Alphelys et al.). Sections were cut and used for IHC.

### Immunohistochemistry

The immunohistochemical staining for VISTA, PDL1, PD1, and CTLA4 expression was performed on a TMA of 135 cores belonging to 135 patients with HGSOC collected between 2000 and 2017 in SAI. The TMA core size was 1mm. Tumor cores of each case from both tissue microarrays were scored independently, and the average score was used.

Tumor sections were assessed immunohistochemically using the following primary monoclonal antibodies (Ab) anti-rabbit: anti-VISTA (Clone ab257314; dilution1/100; pH6; Abcam); to detect the expression of VISTA in both tumoral cells (TCs) and immune cells (ICs). The considerate staining was cytoplasmic and membranous. Anti-PDL1 (clone22C3; Dako) andanti-PD1 (Clone CAL20, prediluted, Abcam) Abs were used to detect the cytoplasmic and membranous expression of PDL1 in both TCs and ICs, and PD1 in only ICs. Anti-human CTLA4 monoclonal Ab (Clone CAU26314; dilution 1/500, BIOMATIK) was used to detect the cytoplasmic and membranous expression of CTLA4 in both TCs and ICs.

Positive expressions in TCs were calculated by summing the number of protein-stained cells, dividing the result by the total number of viable TCs, and multiplying the quotient by 100.

Positive expressions ICs were considered the percentage of tumor-infiltrating ICs (including dendritic cells, macrophages and lymphocytes) in the tumor mass periphery and the stromal bands dissecting the tumor mass at any intensity. The percentage was calculated by dividing the number of protein-stained ICs by the total number of ICs and multiplying the quotient by 100.

Human placenta tissues obtained from the Department of Pathology of SAI were used as a positive control for PDL1/PD1 and VISTA expressions. Tonsil tissues were used as a positive control for CTLA4 expression, and normal ovarian tissues were used as a negative control.

TILs were evaluated using labeling by the following mouse monoclonal antibodies: CD8 (NCL-L-CD8 clone 4B11,1:50, pH9, Novocastra); CD3 (NCL-L-CD3-565, clone LN10, 1:500, pH6, Novocastra); CD4 (NCL-L-CD4-368, clone 4B12, 1:100, pH9, Novocastra), CD56 (Ou NCL-L-504, Clone66556, 1:400, Novocastra), and FOXP3 [clone 236A(E7)], 1:400, pH, Bioscience). The percentage ofCD8^+^, CD3+, CD4^+^, CD56^+^, and FOXP3^+^ lymphocytes compared with the nucleated cells in the stromal and intra-tumoral compartments were assessed.

### IHC scoring

Two experienced ovarian pathologists (RD and GS) analyzed the stained slides using light microscopy. They reviewed the immunohistochemical staining and scored for each sample. The consensus of the two observers was more than 90%. Less than 10% of the sections had inconsistent results, resolved via the joint evaluation of the particular tumor area.

PDL1/PD1 expressions were evaluated with binary positive/negative scoring: PDL1/PD1 positivity was defined as membranous/cytoplasmic staining on ≥1% of the cells using the previously described score (12), which was calculated by summing the number of PDL1/PD1 stained cells (TCs, ICs: lymphocytes and macrophages), diving the result by the total number of cells, and multiplying the quotient by 100.

CTLA4 expression was evaluated using the previously described score (13) based on the intensity of staining and the estimated percentage of positive tumor cells.

Intensity 0: no reaction in cytoplasm, 1^+^: low number of cytoplasmic granules, 2^+^: moderate number of cytoplasmic granules, and 3^+^: if a high number of cytoplasmic granules.

Score 0: 100% of cells with intensity 0 (expression: negative).

Score 1a: <50% of cells with intensity 1^+^ (low-positive), Score 1b:<50% of cells with intensity 2^+^ and/3^+^ (low positive), Score 2a: ≥50% of cells with intensity 1+ (positive); Score 2b: ≥50% of cells with intensity 2^+^ and/3^+^ (positive).

VISTA expression was evaluated in both TCs and ICs. Our previous study described them as positive if at least 1% of these cells per histospot had membranous and cytoplasmic staining (14).

For stratification and statistical analysis purposes, PDL1, PD1, CTLA4, and VISTA expressions were positive if any staining was visible in the TCs or ICs.

The percentage of CD3^+^, CD4^+^, CD8^+^, FOXP3+, and CD56^+^ cells compared with that of the nucleated cells in intratumoral compartments were assessed. The percentages were investigated as continuous values and were dichotomized into low and high groups based on a median of the proportion of these TILs stratified into ‘low’ and ‘high’ groups based on staining scores (corresponding to the median value) 3%, 1%, 3%, 1%, and 1%, respectively, for each core, according to the degree of cell densities. TILs were evaluated according- to the recommendation of the International TILs Working Group 2014 (15).

### TCGA data analysis for mRNA expression

We analyzed VISTA, CTLA4, PD1,PDL1,TILs CD8^+^, CD4^+^, and FOXP3^+^ mRNA expressions in the ovarian carcinoma from the TCGA database (https://www.cancer.gov/ccg/research/genome-sequencing/tcga).

We included 429 samples with the next-generation sequencing (NGS) data (taking into account the Transcripts Per Million (TPM) normalization). All gene expression data were collected and combined in a single file and used to represent the level of correlation or randomness between the multiple expressions as variables.

To investigate the relationship between gene expressions, we used the R package *ggpubr* (v0.6.0) with the *eggs-scatter* function to draw scatter plots between encoding gene *Vsir* (VISTA) and the other genes of interest *CD4*(CD4), *CD8A* (CD8), *FOXP3*(FOXP3), *PDCD1*(PD1), *CD274*(PDL1), and *CTLA4*(CTLA4). Using the Pearson correlation, we used the *stat_cor* function to add significance levels and correlation coefficients. A confidence interval and a regression line were included to highlight the distribution. Each graph indicates the values of R and p-values.

### Statistical analysis

Chi-square test or Fisher’s exact test, displayed by cross-table, were used when appropriate to analyze associations between VISTA, CTLA4, PDL1, and PD1 protein expression, different clinical pathological variables, and TILs (CD3^+^, CD4^+^, CD8^+^, CD65^+^, and FOXP3^+^). A Correlation test (Pearson chi-square test) was used to analyze the correlation between variables. The Kaplan-Meier method was used to depict the survival curves of 2-year OS, and survival curves were compared using the Log-rank test. Univariate and multivariate analyses were based on the Cox proportional hazard, linear and Binary logistic regression models. All data were analyzed with SPSS 22 software. Statistical significance was defined as a *p*<=0.05.

## Results

### Patient clinicopathological characteristics

In the present study, the median age of patients at diagnosis was 55 (range:21-85). Ninety-two patients (68.7%) were younger than 60 years old. One hundred and five patients had advanced stage (III-IV) (77.8%) and 43 (39.1%) with lymph node involvement. Only 32 patients (34.4%) had complete debulking, and only 27 (28.4%) received chemotherapy neoadjuvant. Seventy-five patients (85,1%) were chemo-sensitive, and only 12 (8.9%) had distant metastasis. Fifty-three patients (42.7%) experienced disease progression. At the end of follow-up, the median OS time was 21 months (range:1-189 months), the median Progression-free survival (PFS) time was 16 months (range:1-85 months), and 49 (57%) had died. The 2-year OS rate was 51.85%, and the 2-year PFS rate was 35.55% (**Supplementary Table 1**).

### VISTA, CTLA4, and PDL1 expressions in HGSOC

**Figure 1:**
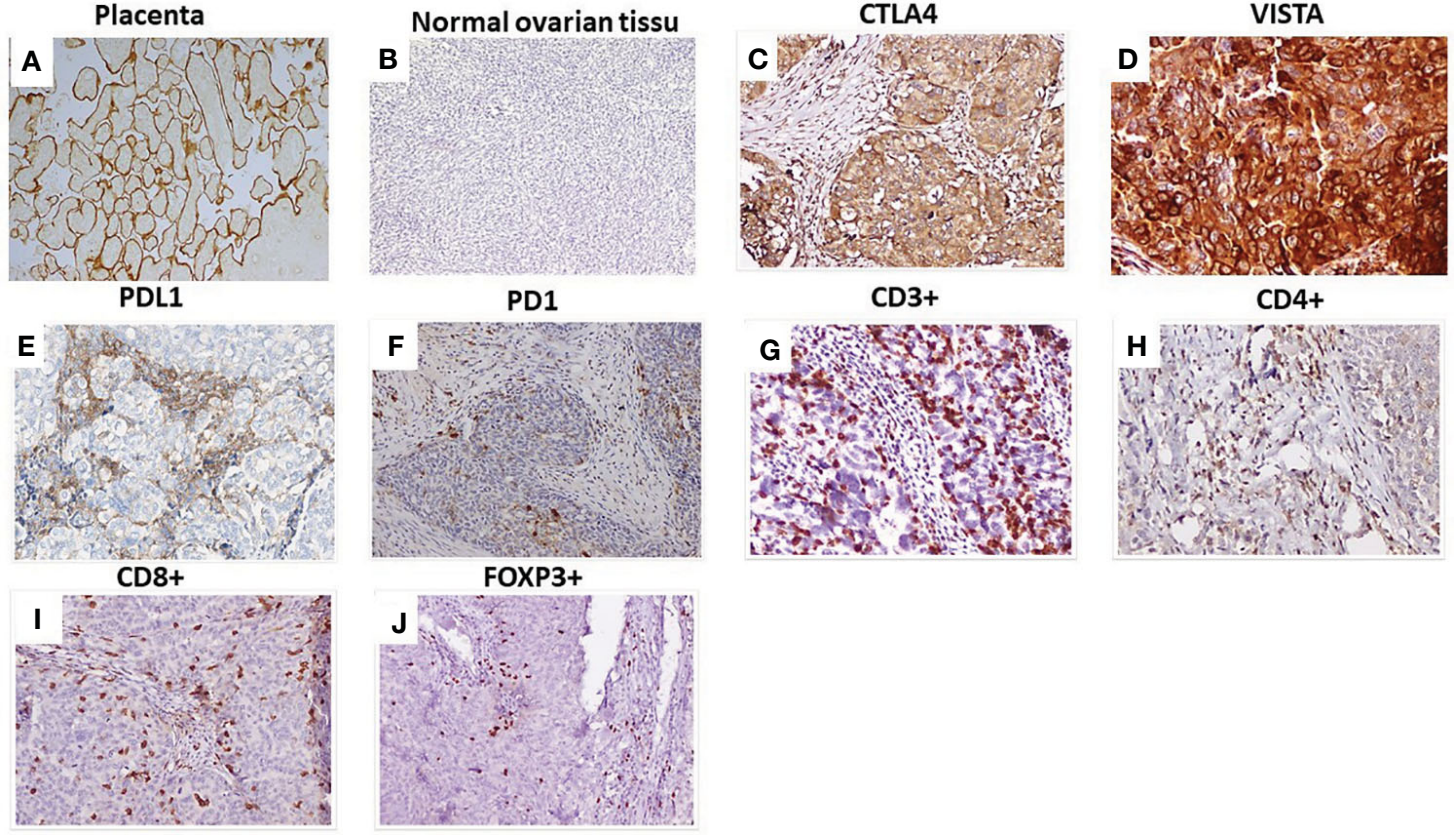
Immunohistochemical staining of checkpoints and tumor-infiltrating lymphocytes in HGSOC. **(A)** VISTA expression in placenta: positive control; **(B)** Normal ovarian tissue: negative control; **(C)** CTLA4 expression in HGSOC; **(D)** VISTA expression in HGSOC; **(E)** PDL1 expression in HGSOC; **(F)** PD1expression in HGSOC. Representative staining densities of tumor-infiltrating lymphocytes expressing: **(G)** CD3; **(H)** CD4; **(I)** CD8 and **(J)** FOXP3 in HGSOC samples. Magnification (×200), scale bare (100 µm). VISTA, V-domain Ig-containing suppressor of T cell activation; CTLA4, Cytotoxic T- lymphocyte- associated protein 4; PD1, Programmed death PD-1; PDL1, Programmed death ligand PDL-1.

Staining using serial sections of HGSOC showed that PD-L1, VISTA, and CTLA4 were expressed in tumor cells and TILs (**Figure 1**). Among the 135 samples, CTLA4 positive staining was detectable in 87.3% (117/135), VISTA was detected in 64.7% (86/135), PD1 was expressed in 56.7% (68/135), and PDL1 was expressed in 48.1% (64/135). Among samples with VISTA positive expression, 77 samples were CTLA4+(89.6%), 53 samples were PD1+(67.1%), and only49 samples were PDL1+(57.6%) (**Table 1**).

**Table 1:** The correlation between VISTA, CTLA4, PDL1 and PD1 expressions and clinicopathological characteristics.

	VISTA expression			CTLA4 expression				PDL1 expression			PD1 expression		
	Positive	Negative	*p*	Low positive	Positive	Negative	*p*	Positive	Negative	*p*	Positive	Negative	
Age (years)													
<60	54 (59.3%)	37 (40.7%)	0.071	24 (26.1%)	54 (58.7%)	14 (15.2%)	0.45	43 (74.3%)	48 (52.7%)	0.871	46 (56.1%)	36 (43.9%)	0.732
>=60	31 (75.6%)	10 (24.4%)		12 (29.3%)	26 (63.4%)	3 (7.3%)		20 (48.8%)	21 (51.2%)		22 (59.5%)	15 (40.5%)	
TNM stage													
Non Advanced	19 (63.3%)	11 (36.7%)	0.863	8 (26.7%)	18 (60.0%)	4 (13.3%)	1	19 (63.3%)	11 (36.7%)	**0,058**	18 (62.1%)	11 (37.9%)	0.5
Advanced	67 (65.0%)	36 (35.0%)		28 (26.9%)	63 (60.6%)	13 (12.5%)		45 (43.7%)	58 (56.3%)		50 (54.9%)	41 (45.1%)	
LN involvement													
Yes	30 (69.8%)	13 (30.2%)	0.301	10 (23.3%)	29 (67.4%)	4 (9.3%)	0.418	22 (52.4%)	20 (47.6%)	0.583	23 (59.0%)	16 (41.0%)	0.895
No	39 (60.0%)	26 (40.0%)		18 (27.3%)	37 (56.1%)	11 (16.6%)		31 (47.0%)	35 (53.0%)		34 (57.6%)	25 (42.4%)	
Chemo neoadj													
Yes	17 (65.4%)	9 (34.6%)	0.979	7 (25.9%)	17 (63.0%)	3 (11.1%)	0.947	16 (61.5%)	10 (38.5%)	0.077	16 (66.7%)	8 (33.3%)	0.211
No	44 (65.7%)	23 (34.3%)		20 (29.9%)	28 (56.7%)	9 (13.4%)		28 (41.2%)	40 (58.8%)		31 (51.7%)	29 (48.3%)	
VISTA													
Positive				17 (19,8%)	60 (69,8%)	9 (10,4%)	**0.008**	49 (57.6%)	36 (42.4%)	**0.005**	53 (67.1%)	26 (32.9%)	**0.001**
Negative				19 (40.4%)	20 (42.6%)	8 (17.0%)		15 (31.9%)	32 (68.1%)		15 (36.6%)	26 (63.4%)	
CTLA4													
Low positive	17 (47.2%)	19 (52.8%)	**0.008**					14 (38.9%)	22 (61.1%)	0.426	12 (35.3%)	22 (64.7%)	**0.002**
Positive	60 (75.0%)	20 (25.0%)						41 (51.9%)	38 (48.1%)		46 (61.3%)	29 (38.7%)	
Negative	9 (52.9%)	8 (47.1%)						9 (52.9%)	8 (47.1%)		10 (90.9%)	1 (9.1%)	
PDL1													
Positive	49 (76.6%)	15 (23.4%)	**0.005**	14 (21.9%)	41 (64.1%)	9 (14.0%)	0.426				47 (81.0%)	11 (19.0%)	**<0.001**
Negative	36 (52.9%)	32 (47.1%)		22 (32.3%)	38 (55.9%)	8 (11.8%)					20 (32.8%)	41 (67.2%)	
PD1													
Positive	53 (77.9%)	15 (22.1%)	**0.001**	12 (17.6%)	46 (67.7%)	10 (14.7%)	**0.002**	47 (70.1%)	20 (29.9%)	**<0.001**			
Negative	26 (50.0%)	26 (50.0%)		22 (42.3%)	29 (55.8%)	1 (1.9%)		11 (21.1%)	41 (78.8%)				

VISTA, Domain Ig suppressor of Tcell activation; CTLA4, cytotoxic T- lymphocyte-associated protein 4; PD1/PDL1, programmed death/ligand; LN, lymph node; TNM, Tumour, Node, Metastasis classification; chemo neoadj, chemotherapy neoadjuvant.

Bold means significant association *p*<=0.05.

### Correlation of VISTA, PDL1, PD1, and CTLA4 expression with the clinicopathological features

As shown in **Table 1**, only PDL1 positive expression was associated with the advanced stage (*p*=0.058). There was no correlation between VISTA, CTLA4, PDL1, PD1, and clinicopathological parameters. VISTA was correlated with CTLA4 expression (*p*=0.008), with PDL1 expression (*p*=0.005), and with PD1 expression (*p*=0.001). Even, CTLA4 expression was associated with PD1 (*p*=0.002) but not PDL1 (*p*=0.426). Also, PDL1 was strongly correlated with PD1 (*p*<0.001) (**Table 1**).

### Correlation of VISTA, CTLA4, PDL1, and PD1 expression with TILs

Given that the immune checkpoints VISTA, CTLA4, PDL1, and PD1 display suppressive effects on TILs. In this study, we evaluated the correlation between these checkpoints and TILs, including CD3^+^, CD4^+^, CD8^+^, CD56^+^, and FOXP3^+^ by IHC (**Figure 1**). As shown in **Table 2**, VISTA expression was correlated with CD4^+^(*p*=0.009), with CD8^+^ (*p*=0.004), and with FOXP3^+^ (*p*=0.037). VISTA-positive expression in HGSOC tumors was more frequent in patients with high CD3^+^ but not significantly correlated (*p*=0.631). The density of CD3^+^, CD4^+^, CD8^+^ and FOXP3^+^ TILs were significantly higher in tumors with PD1 positive expression than that in corresponding PD1 negative expression (*p*=0.021; *p*<0.001; *p*<0.001; *p*<0.001, respectively). PDL1 expression was strongly correlated with TILs CD4^+^ (*p*<0,001), CD8^+^ (*p*=0,003) and FOXP3 (*p*=0.006). CTLA4 tends to be correlated with TILs (CD3*p*=0.061, CD4^+^ *p*=0.077, CD8 *p*=0.086, and associated with FOXP3^+^ *p*=0.007). In contrast, there was no correlation with CD56^+^ (**Table 2**). These data showed positive expressions of immune checkpoints VISTA, CTLA4, PDL1, and PD1 were associated with TIL infiltration in HGSOC.

**Table 2:** Correlation between VISTA, PD1, PDL1, CTLA4 expression and tumor infiltrating lymphocytes.

TILs	VISTA			CTLA4				PDL1			PD1		
	Positive	Negative	*P*	Positive	Low Positive	Negative	*P*	Positive	Negative	*P*	Positive	Negative	*P*
CD3^+^ (≥3%)	54 (66.7%)	27 (33.3%)	0.631	17 (21.0%)	54 (66.7%)	10 (12.3%)	**0.061**	41 (50.6%)	40 (49.4%)	0.599	47 (66.2%)	24 (33.8%)	**0.021**
CD3^+^ (<3%)	30 (62.5%)	18 (37.5%)		19 (38.8%)	23 (46.9%)	7 (14.3%)		22 (45.8%)	26 (54.2%)		20 (44.4%)	25 (55.6%)	
CD4^+^ (≥1%)	57 (76.0%)	18 (24.0%)	**0.009**	16 (21.3%)	52 (69.4%)	7 (9.3%)	**0.077**	49 (66.2%)	25 (33.8%)	**<0.001**	57 (78.1%)	16 (21.9%)	**<0.001**
CD4^+^ (<1%)	19 (51.4%)	18 (48.6%)		15 (40.5%)	18 (48.6%)	4 (10.8%)		7 (18.9%)	30 (81.1%)		9 (24.3%)	28 (75.7%)	
CD8^+^ (≥3%)	51 (77.3%)	15 (22.7%)	**0.004**	13 (19.7%)	46 (69.7%)	7 (10.6%)	**0.086**	40 (61.5%)	25 (38.5%)	**0.003**	52 (80.0%)	13 (20.0%)	**<0.001**
CD8^+^ (<3%)	26 (52.0%)	24 (48.0%)		19 (38.0%)	28 (56.0%)	3 (6.0%)		17 (34.0%)	33 (66.0%)		14 (29.8%)	33 (70.2%)	
Foxp3 (≥1)	38 (79.2%)	10 (20.8%)	**0.037**	5 (10.4%)	38 (79.2%)	5 (10.4%)	**0.007**	32 (68.1%)	15 (31.9%)	**0.006**	38 (82.6%)	8 (17.4%)	**<0.001**
Foxp3 (<1)	35 (60.3%)	23 (39.7%)		21 (36.2%)	31 (53.4%)	6 (10.4%)		24 (41.4%)	34 (58.6%)		28 (49.1%)	29 (50.9%)	
CD56^+^ (≥1)	11 (68.8%)	5 (31.3%)	0.952	2 (12.5%)	12 (75.0%)	2 (12.5%)	0.209	8 (47.1%)	9 (52.9%)	0.793	10 (62.5%)	6 (37.5%)	0.838
CD56^+^ (<1)	68 (68.0%)	32 (32.0%)		32 (32.0%)	60 (60.0%)	8 (8.0%)		50 (50.5%)	49 (49.5%)		58 (59.8%)	39 (40.2%)	

VISTA, Domain Ig suppressor of Tcell activation; CTLA4, cytotoxic T- lymphocyte- associated protein 4; PD1/PDL1, programmed death/ligand; TILs, Tumor infiltrating lymphocytes.

Bold means significant association *p*<=0.05.

**Figure 2:**
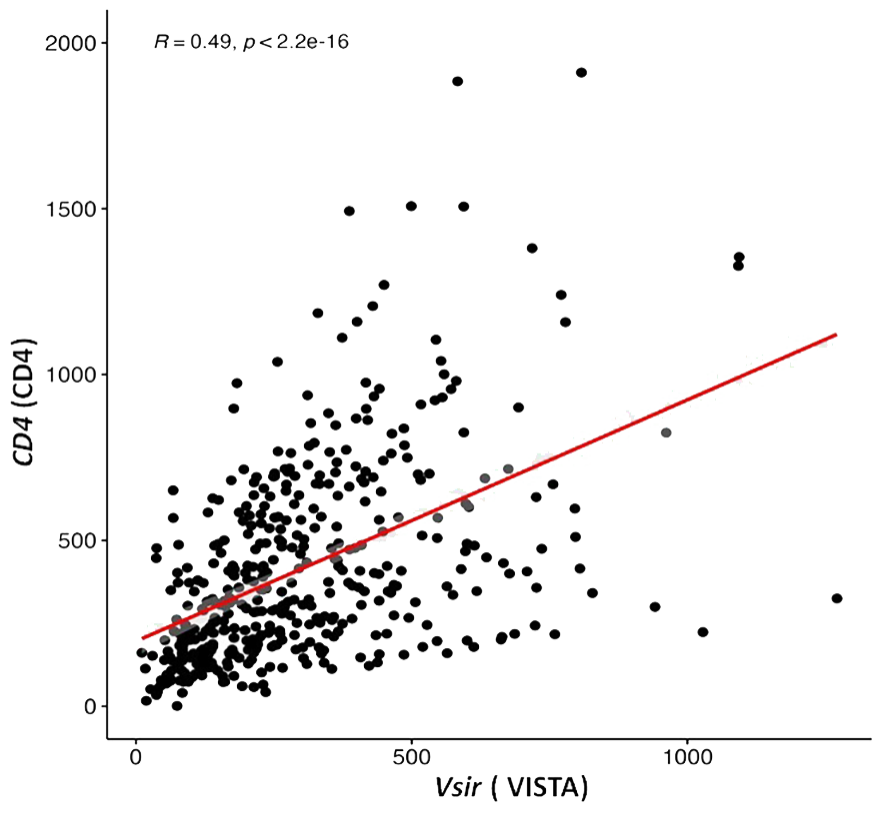
V-domain Ig-containing suppressor of T cell activation (VISTA) encoding gene is correlated with expression of a gene that encodes CD4 in ovarian cancer samples from the Cancer Genome Atlas public database (TGCA).

To further evaluate the relevance of VISTA, CTLA4, PD1and PDL1 expressions and TILs markers in HGSOC at the mRNA level, we assessed the correlation between the *Vsir* gene (encoding VISTA), *CTLA4*(encoding CTLA4), *CD274*(encoding PDL1), *PDCD1* (encoding PD1), *CD8A* (encoding CD8), *CD4* (encoding CD4) and *FOXP3* (encoding FOXP3) according to the mRNA expression of 429 ovarian cancer patients downloaded from the TCGA database. Although the gene encoding VISTA was positively associated with genes encoding CTLA4 (*p*<0.01, R=0.21), PDL1 (*p*<0.01, R=0.17), PD1 (*p*<0.01, R=0.26), CD8 (*p*<0.01, R=0.26), and FOXP3 (*p*<0.01, R=0.26), the correlations were weak (R<0.4) (**Supplementary Figure 1**). However, a positive correlation was found between VISTA and CD4^+^ encoding genes (*p*<0.01, R=0.49) (**Figure 2**).

### Prognostic values of VISTA, CTLA4, PDL1, and PD1in HGSOC in terms of OS

**Figure 3:**
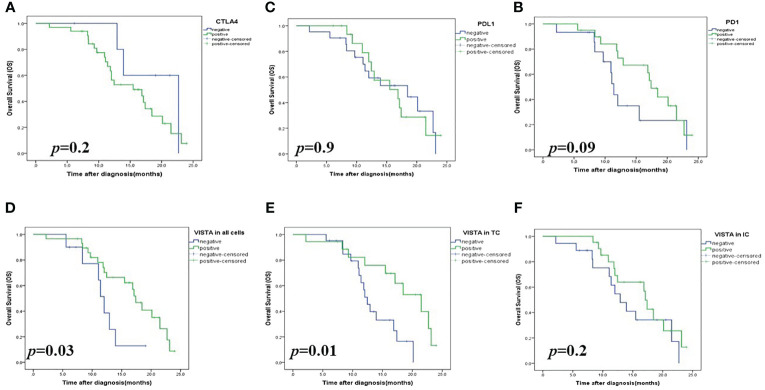
Kaplan-Meier survival analysis (OS) of checkpoint expressions in patients with High-Grade Serous Ovarian Carcinoma. **(A)** CTLA4 expression in all cells; **(B)** PDL1 expression in all cells; **(C)** PD1 expression in all cells; **(D)** VISTA expression in all cells; **(E)** VISTA expression on tumors (TCs); **(F)** VISTA expression on immune cells (ICs). VISTA, V-domain Ig-containing suppressor of T cell activation; CTLA4, Cytotoxic T- lymphocyte- associated protein 4; PD1, Programmed death PD-1; PDL1, Programmed death ligand PDL-.

In terms of 2-year OS, Kaplan-Meier analysis results showed that a significant association was found between OS and VISTA expression (*p=*0.03) but not correlated with PDL1 (*p*=0.9), PD1 (*p*=0.09), and CTLA4 (*p*=0.2) (**Figure 3**). Univariate analyses showed that only VISTA expression (*p*=0.04) was correlated with 2-year OS. Multivariate analyses showed that VISTA expression remained the only independent significant factor for 2-year OS (*p*=0.01) (**Table 3**).

**Table 3:** Uni and multivariate analyses of prognostic factors correlated with two years of0verall survival.

	OS			
	Univariate analyses		Multivariate analyses	
Checkpoints	HR (95%)	*p*	HR (95%)	*p*
VISTA expression	0.39 (1.55-9,88)	**0.04**	0.21 (0.06-0.72)	**0.01**
PDL1 expression	0.98 (0.44-2.25)	0.97	–	–
PD1 expression	0.48 (0.20-1.16)	0,1	0.90 (0.30-2.69)	0.86
CTLA4 expression	1.51 (0.76-3.05)	0.22	1.81 (0.77-4.27)	0.17

OS, Overall survival; VISTA, V domain Ig suppressor of Tcell activation; PD1/PDL1, programmed death/ligand; CTLA4, cytotoxic T- lymphocyte-associated protein 4; HR, Hazard ratio.

Bold means significant association *p*<=0.05.

### VISTA expression on tumor cells reveals long-term survival in HGSOC patients

In the Kaplan Meier curves analysis, patients with a high VISTA expression showed a significant difference in 2-year OS compared to those with low VISTA expression (*p=*0.03).

Next, we explored whether VISTA-positive cell types affect the prognosis of patients with HGSOC.As a result, patients with VISTA-positive staining in TCs (*p*= 0.01) but not in tumor-infiltrating ICs (*p*= 0.2) showed significantly prolonged OS compared to those with negative VISTA expression (44% vs 28.6%) (**Figure 3**). These data suggested a favorable survival of patients with HGSOC with VISTA staining in TCs.

### Characterization of the immune microenvironment based on VISTA, CTLA4, PDL1, and PD1

Our study initially categorized the patients as VISTA/PDL1, VISTA/PD1, VISTA/CTLA4, CTLA4/PD1, and CTLA4/PDL1. Then, patients were classified by a combination of the three checkpoints: VISTA/CTLA4/PDL1 or VISTA/CTLA4/PD1.

**Figure 4:**
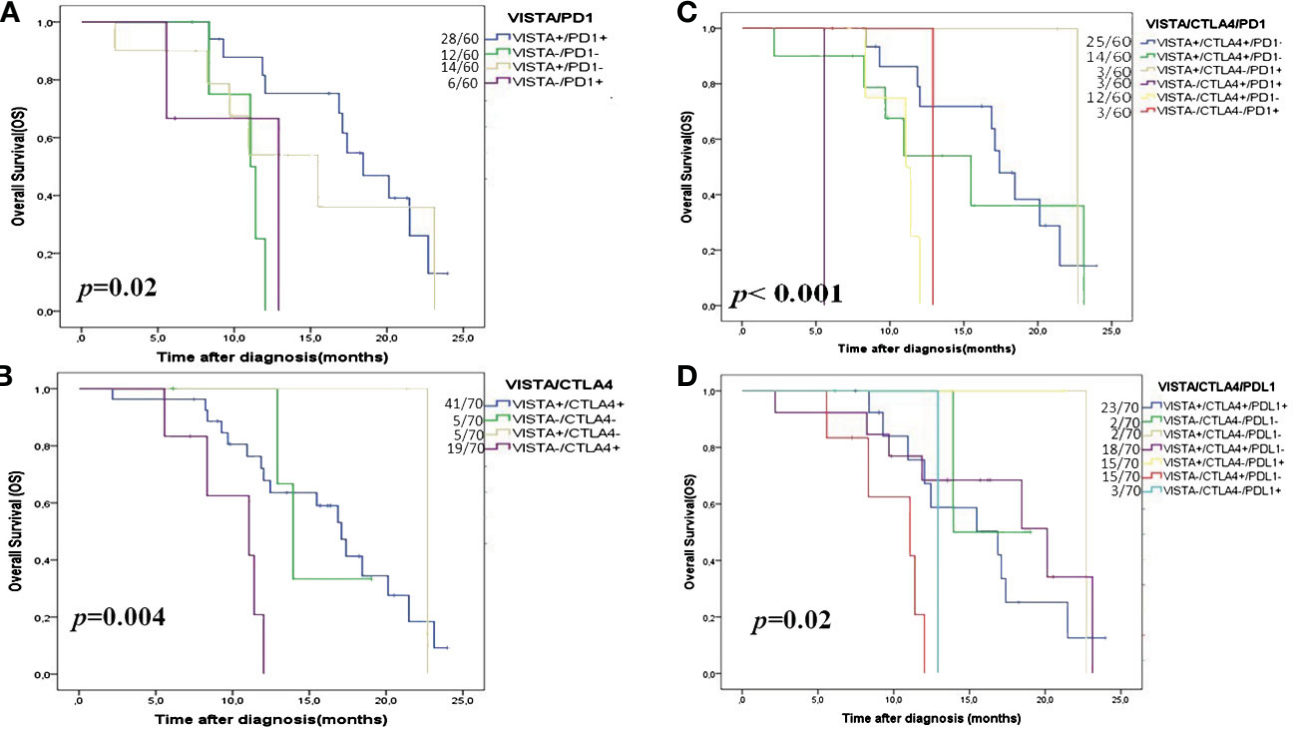
Kaplan- Meier OS curves in HGSOC according to double and triple immune checkpoint expressions. **(A)** VISTA/PD1; **(B)** VISTA/CTLA4; **(C)** VISTA/CTLA4/PD1 and **(D)** VISTA/CTLA4/PDL1. VISTA is a V-domain Ig-containing suppressor of T cell activation; CTLA4 is cytotoxic T-lymphocyte-associated protein 4; PD1 is programmed death PD-1, and PDL1 is programmed death ligand PDL-1.

Survival analyses demonstrated that only patients with VISTA^+^/PD1^+^ (66.7%) and VISTA^+^/CTLA4^+^ (91.8%) were associated with longer 2-year OS (*p*=0.02 and *p*=0.004, respectively) (**Figure 4**; **Supplementary Figure 2**).

Using multivariate analyses combining checkpoints with OS we tried to evaluate which combination had the strongest association with 2-year OS. After adjusting for possible confounding variables, multivariate analyses showed that the combination VISTA/PD1, VISTA/CTLA4, and VISTA/CTLA4/PD1 remained the significant independent factors for 2-year OS (*p*=0.005, *p*=0.01, and *p*=0.05, respectively) (**Table 4**).

**Table 4:** Uni and multivariate analyses of checkpoints coexpression correlated with OS.

	OS			
	Univariate analyses		Multivariate analyses	
Checkpoints coexpression	HR (95%)	*p*	HR (95%)	*p*
VISTA/PDL1	0.94 (0.62-1.45)	0.77	–	
VISTA/PD1	1.46 (0.98-2.19)	**0.06**	3.89 (1.49-10.15)	**0.005**
VISTA/CTLA4	1.35 (0.92-1.97)	0.11	4.02 (1.38-11.69)	**0.01**
CTLA4/PDL1	0.98 (0.66-1.44)	0.93	–	
CTLA4/PD1	1.06 (0.75-1.48)	0.72	–	
VISTA/CTLA4/PDL1	1.14 (0.93-1.39)	0.19	(0.91 (0.65-1.27)	0.6
VISTA/CTLA4/PD1	1.16 (0.97-1.40)	**0.09**	0.53 (0.28-1)	**0.05**

OS, Overall survival; VISTA, V domain Ig suppressor of Tcell activation; PD1/PDL1, programmed death/ligand; CTLA4, cytotoxic T- lymphocyte-associated protein 4; HR, Hazard ratio.

Bold means significant association *p*<=0.05.

Interestingly, Kaplan-Meier analysis showed that the coexpression of the three checkpoints (VISTA/CTLA4/PDL1 and VISTA/CTLA4/PD1) had a significant correlation with 2-year OS (*p*=0.02 and *p*<0.001, respectively) (**Figure 4**). Finally, Cox regression analysis of the HGSOC cohort showed that in the synergic expression VISTA/CTLA4/PD1, VISTA expression on TCs refers to the prolonged OS (**Table 5**).

**Table 5:** VISTA expression in tumor cells refers to better prognostic.

	OS			
	Univariate analyses		Multivariate analyses	
Coexpression	HR (95%)	*P*	HR (95%)	*P*
VISTATC/CTLA4/PD1	1.21 (1.02-1.43)	**0.02**	1.20 (1.01-1.42)	**0.03**
VISTAIC/CTLA4/PD1	1.10 (0.92-1.30)	0.26	1.05 (0.88-1.26)	0.54

OS, Overall survival; VISTA, V domain Ig suppressor of Tcell activation; CTLA4, cytotoxic T- lymphocyte- associated protein 4; PD1/PDL1, programmed death/ligand; TC, Tumor cells; IC, Immune cells; HR, Hazard ratio.

Bold means significant association *p*<=0.05.

### Characterization of the immune microenvironment according to VISTA^+^/CTLA4^+^/PD1^+^and TILs

The VISTA^+^/CTLA4^+^/PD1^+^ (37.98%) group has the best prognosis, so we must look for lymphocyte infiltration associated with this positive expression. Univariate analysis showed that CD3^+^ (*p*=0.01), CD4^+^ (*p*<0.001), CD8^+^ (*p*<0.001), and FOXP3^+^ (*p*=0.001) TILs are strongly correlated with the synergistic and positive expression of the three checkpoints. However, the multivariate analysis proved that only CD4^+^ (58.7%) and CD8^+^ (56.5%) TILs (*p*=0.008) remain independent factors for the positive coexpression of the VISTA/CTLA4/PD1 checkpoints (**Table 6**).

**Table 6:** Univariate and Multivariate analyses of TILs correlated with VISTA^+^/CTLA4^+^/PD1^+^.

VISTA^+^/CTLA4^+^/PD1^+^				
TILs	Univariate analyses		Multivariate analyses	
	HR (95%)	*P*	HR (95%)	*P*
CD3^+^	0.45 (0.24-0.84)	0.01	1.07 (0.47-2.44)	0.86
CD4^+^	0.08 (0.02-0.28)	<0.001	0.16 (0.04-0.62)	0.008
CD8^+^	0.22 (0.10-0.49)	<0.001	0,26 (0.10-0.70)	0.008
CD56^+^	0.77 (0.29-2.08)	0.61	–	‘-
FOXP3^+^	0.24 (0.10-0.55)	0.001	0.59 (0.21-1.16)	0.3

VISTA, V domain Ig suppressor of Tcell activation; CTLA4, cytotoxic T- lymphocyte- associated protein 4; PD1/PDL1, programmed death/ligand; TILs, Tumor infiltrating lymphocytes; HR, Hazard ratio.

Finally, we wanted to look for a favorable tumor microenvironment model that influences the OS in our study. By multiple linear regression test, we found that the TME was rich with both TILs: CD4^+^, CD8^+^, FOXP3^+^, and coexpressed checkpoints VISTA/CTLA4/PD1 was correlated with a favorable prognosis (*p*=0.04; R=0.4) (**Table 7**).

**Table 7:** Tumor microenvironment model correlated with Overall survivalin HGSOC.

TME	*P*	R
VISTA/CTLA4/PD1, CD8+, CD4+, FOXP3+	**0.04**	0.42
VISTA: CTLA4/PDL1, CD8+, CD4+, Foxp3+	0.1	0.38

VISTA, Vdomain Ig suppressor of Tcell activation; CTLA4, cytotoxic T- lymphocyte- associated protein 4; PD1/PDL1, programmed death/ligand; TME, Tumor microenvironment; HGSOC, High-grade serous carcinoma.

Bold means significant association *p*<=0.05.

## Discussion

VISTA has attracted broad interest as a novel immune checkpoint that suppresses the activity of T cells (16). However, little is known about its expression profile in EOC. Recent studies have shown that VISTA expression has increased after PD1 blockage in metastatic melanoma (17, 18) and CTLA4 in prostate cancer (19). These results indicated that VISTA may play a significant role in immunotherapy resistance (20, 21).

In the present work, our objectives were to explore the distribution of the immune checkpoints VISTA, PDL1, PD1, and CTLA4 in the TME, their correlation with the clinicopathological features, TILs, and their prognostic value in an extensive series of HGSOC. Then, the mRNA expressions of the immune checkpoints were extracted from 429 patients with ovarian cancer in the TCGA database and analyzed. This research showed thatPDL1, PD1, CTLA4, and VISTA were variably expressed in all cells (including TCs, ICs, and endothelial cells) in HGSOC. A previous study showed that PDL1 was strongly expressed in gestational trophoblastic neoplasia (22) (GTN), (23), in 8,9% of EOC (8), and 33% of HGSOC (24). At the same time, Wang and colleagues showed that PDL1 was expressed in 24.3% of HGSOC (25).

In terms of prognosis, PDL1 expression remains controversial. In the literature, Jo et al. found that PDL1 expression was associated with a prolonged OS in Extranodal Natural Killer/T-cell Lymphoma (ENKTCL) (17) and breast cancer (26, 27). However, PDL1 expression was associated with shorter OS in pancreatic cancer, hepatocellular carcinoma and gastric cancer (26, 28), and no association was observed between PDL1 expression and survival in patients with EOC (8, 24). Our study showed an expression of PDL1 in 48.1% of HGSOC associated with an advanced stage and not correlated to 2-year OS in HGSOC.

Concerning PD1, the protein expression was detected in 56.7% of HGSOC and was expressed only in ICs. In the literature, the authors did not find any association between PD1 expression and OS (24, 29), which is consistent with our findings in HGSOC. Furthermore, in this work, CTLA4 expression was highly expressed (87.3%), compared with PDL1 and PD1, but was not associated with OS. In a recent study, after a systematic investigation of 50 immune checkpoint genes, Fang et al. found that high expression of CTLA4 was associated with a better prognosis in breast cancer (29).

We further analyzed VISTA expression in HGSOC and found its expression in 64.7% of HGSOC. Previous studies showed that VISTA was expressed in 29.5% of hepatocellular carcinoma, 51.4% of HGSOC, 25.6% of pancreatic cancer cells in 100% of endometrial cancer, and 99% of lung cancer (3, 6, 8, 30). VISTA has recently been identified as a potent suppressor of T activation, which produces a poor prognosis in theory. However, VISTA expression and its relationship with patient survival vary according to the cancer type. In the literature, it was correlated with poorer prognosis in prostate cancer (19), acute myeloid leukemia (AML) (31), breast cancer (32), and melanoma (33). In contrast, VISTA correlated with a favorable prognosis in TNBC (34), colorectal cancer (35), hepatocellular carcinoma (6), pancreatic cancer (36), and in HGSOC (8), but no association between VISTA expression and OS in ovarian cancer (7, 36) and GTN (23)was found.

Our previous study (14) evaluated VISTA in EOC and included all histological types (15). We showed that VISTA was expressed in both TCs and ICs but had no correlation with OS. However, in this study, we stratified our population and evaluated the expression of VISTA in the HGSOC histological type according to all cells, TCs or ICs. We showed a high expression of VISTA in TCs, which was significantly correlated with a better prognosis in patients with HGSOC. These results show that VISTA’s expression largely depends on the tumor type. Here, our findings could be partly explained by the fact that VISTA plays the role of a ligand when expressed on lymphocytes T and antigen-presenting cells (APC) and only a receptor on LT (4, 37). Its expression on the tumor cells would make it a ligand that inhibits the T cells’ activation. Thus, we suggest that in HGSOC, the VISTA receptor role on TCs could be related to its association with better prognosis by affecting the tumor cell itself following its interaction with its ligand expressed in the TME.

These data suggest that VISTA expression in TCs and ICs can perform different functions via distinct mechanisms. This ambivalent role of VISTA should be considered in immunotherapy using anti-VISTA antibodies.

Then, we wanted to analyze whether the coexpression of the immune checkpoints has a prognostic value in HGSOC. We found that VISTA was highly coexpressed with CTLA4, PDL1, and PD1 in HGSOC. The positive correlation between VISTA, PDL1, and PD1 expressions was in agreement with the literature in non-small cell lung cancer (NSCLC), ENKTCL, HGSOC, breast cancer, gastric cancer, oral squamous cell carcinoma, and epithelioid malignant pleural mesothelioma (5, 8, 17, 30, 32, 38–40).

To further validate our results, we analyzed the correlation between VISTA, CTLA4, PDL1, and PD1 mRNA expressions based on TCGA analysis. The results showed a weak correlation between genes *Vsir* (VISTA) and *CTLA4*(CTLA4), *Vsir* and *CD274*(PDL1), *Vsir*and*PDCD1* (PD1), *Vsir and CD8A* (CD8), as well as *Vsir and FOXP3*(FOXP3). In contrast, the correlation between *Vsir and CD4* (CD4) was significant. The protein and mRNA expression results further supported the possibility that combining VISTA, CTLA4, and PDL1/PD1 blockade might be a promising option to overcome checkpoint inhibitor resistance and elicit synergistic effects in stimulating anti-tumoral immune responses.

In the literature, VISTA, CTLA4, and PDL1/PD1 facilitate the immune escape via separate inhibitory pathways (17, 41). Several studies showed that upon PD1/PDL1 or CTLA4 blockade, an upregulation of VISTA was induced, which may suggest that VISTA could contribute to immune checkpoint blockade resistance through different mechanisms modulated by intracellular signaling pathways and the TME modulation, in which locally secreted factors such as interleukins or interferons could mediate VISTA upregulation (18, 42).

Mulati et al. reported that an anti-VISTA (Ab) prolonged the survival of mice with ovarian tumors (3). In the present study, only VISTA in a single expression was correlated with prolonged OS among the other immune checkpoint expressions (CTLA4, PD1 or PDL1) in- HGSOC patients. Therefore, this study examined the effect of the checkpoint’s coexpression two by two on OS. Interestingly, we demonstrated that VISTA/PD1 and VISTA/CTLA4 coexpression were associated with prolonged 2-year OS in HGSOC. From the literature, in a recent study, authors showed that a combination of VISTA and PD1 blockade achieved optimal tumor-clearing therapeutic efficacy in the double Knockout (KO)colon cancer mice models (9).

To our knowledge, our study is the first to evaluate the prognostic value of the three immune checkpoints: VISTA, CTLA4, and PDL1/PD1. For better stratification, we also classified patients into two immune groups, VISTA/CTLA4/PDL1 or VISTA/CTLA4/PD1. Interestingly, multivariate analyses showed that the combination of VISTA^+^/CTLA4^+^/PD1^+^ was an independent predictor of prolonged 2-year OS. The Kaplan Meier analyses showed positive VISTA/CTLA4/PDL1 and VISTA/CTLA4/PD1combinationscorrelated with prolonged 2-year OS. Using Cox regression analysis, we showed that the VISTA expression on TCs was related to this better prognostic when coexpressed with other immune checkpoints, and patients with VISTA-positive expression had a prolonged OS even when CTLA4 or PD1 were negative. The published data shows that combination therapy with a VISTA antagonist is more efficient than an immune checkpoint in a single therapy. Combined CTLA4 and VISTA blockade treatment was more efficient than PD1 and VISTA blockade in head and neck squamous cell carcinoma models (43).

Our study also found a positive correlation between VISTA, CTLA4, PDL1, PD1checkpoints, and TILs, which was confirmed by a recent study in HGSOC (44) in colorectal cancer (45), TNBC (34), and hepatocellular carcinoma (6). The previously reported induction of VISTA and PD-L1 by pro-inflammatory cytokines such as IFN-γ could mechanistically support the markers’ coexpression and the positive association with TILs.

Furthermore, in the present research study, multivariate analyses showed that only CD8^+^ and CD4^+^ remain the independent TILs associated with the checkpoints combined expression VISTA/CTLA4/PD1, and the mean counts of CD8^+^ and CD4^+^ TILS were higher in patients with VISTA^+^/CTLA4^+^/PD1^+^ positive expression, which suggests a paracrine mechanism used by cancer cells to communicate with the immune system by the expression of these negative checkpoints, as reported for PD-L1 in breast cancer patients (46, 47). IFN-γ prominently upregulated VISTA, PDL1and PD1.In the tumor microenvironment, CD8^+^ T cells are the main IFN-producing immune cells after a signal from CD4^+^ (48, 49). We can suggest that there is negative feedback in the immunomodulatory mechanism between these immune checkpoints, CD4^+^ and CD8^+^ TILs in HGSOC. Cytotoxic T lymphocytes (LT-CD8) supported by LT-CD4 (TH1) are potent effectors of tumor cell elimination via IFN secretion. In turn, VISTA and PD1 expression inhibit the activation and the proliferation of TILs and undermine antitumor immune response in HGSOC. Moreover, recent *in vivo* studies showed that VISTA exerts a quiescent function on naive LTs, inducing their inactivation. However, these effects were lost on specific LT under inflammatory conditions where the effect of VISTA was downregulated or attenuated (32, 50).

We also suggest that in the inflammatory microenvironment, there are molecules or inflammatory cytokines that block the inhibitory effect of different immune checkpoints such as VISTA, CTLA4, PDL1, and PD1 or modify the pH of the TME to become unfavorable for the inhibitory action of these checkpoints on lymphocytes T. In this case, the combination of VISTA, CTLA4, and PD1checkpoint blockade was associated with an essential density of TILs compared to using either checkpoint in single or double treatment.

Finally, we created a TME model that influences the 2-year OS to validate our previous results further. Using multiple linear regression tests, we found that the simultaneous expression of CD4^+^, CD8^+^, Foxp3^+^ TILs and the checkpoints coexpression VISTA/CTLA4/PD1 predict the prolonged 2-yearOS with the dominance of the simultaneous expression VISTA/CTLA4/PD1 as an independent predictor of 2-year OS. In our previous study, in a whole series of EOC patients (14), we found that the patients with VISTA^+^/CD8^+^ had a prolonged OS.

Overall, our data suggest that an increased immune cell infiltration may be insufficient to generate antitumor responses, and combined blockade of the immune checkpoints VISTA, CTLA4, and PD1 may be necessary to provide longer OS for patients with HGSOC. These results suggest synergistic VISTA, CTLA4, and PD1blockade can enhance antitumor immunity, suppress tumor growth by enhancing CD4^+^and CD8^+^ TILs in the TME and overcome immune checkpoints inhibitory resistances.

Therefore, the combination blockade of VISTA/CTLA4/PD1 may be an efficient combined therapy. This would simultaneously focus on releasing multiple breaks and induced CD8^+^ and CD4^+^ T cell activation by converting resting and exhausted cells into functional effector cells for a more potent immune response for patients with HGSOC.

Furthermore, the underlying molecular mechanism of VISTA/CTLA4/PD1 in combination therapy should be explored in HGSOC as biomarkers for prognosis *in vivo* and clinical translation. The ambivalent role of VISTA should be considered in immunotherapy using anti-VISTA antibodies that should not block VISTA as a receptor on TCs.

From the previous studies and our present findings, we hypothesize that VISTA could be a receptor on HGSOC tumor cells and that should be evaluated in preclinical studies. Single and coexpression of VISTA TCs/CTLA4/PD1 in HGSOC, with their immune inhibitory capability, suggests that these immune checkpoints could be a potential novel target for immunotherapy against HGSOC. Thus, a better understanding of the VISTA, CTLA4, and PD1 expression and coexpression *in vitro* and *in vivo* models could reveal new prognostic biomarkers and improved options for immunotherapy in patients with HGSOC.

In conclusion, our study revealed that VISTA expression was associated with CTLA4, PDL1 and PD1 expressions in the HGSOC cohort. The single expression of these checkpoints displayed prognostic diversity. VISTA had cell-specific and prognostic diversity in HGSOC. Furthermore, we showed that the combination of VISTA^+^/CTLA4^+^/PD1^+^ was an independent predictor of prolonged 2-year OS, and the expression of VISTA in tumor cells refers to this association with a favorable prognosis in patients with HGSOC. VISTA/CTLA4/PD1 coexpression was closely correlated with TILs. Despite these findings, our study had several limitations, including those inherent to a retrospective study. First, given the intratumoral heterogeneity, TMA may not have accurately represented the entire tumor regarding marker expression. Second, the follow-up time of the validation cohort was relatively short. Altogether, this work established the significance of VISTA/CTLA4/PD1 coexpression in the prognosis and immune microenvironment of HGSOC. Further studies should explore VISTA’s ambivalent role in preclinical studies, as this will provide more options for HGSOC immunotherapy.

## Data availability statement

Data contains potentially identifying and sensitive patient information (patients' ID and Date of Birth). Data restrictions were set by the Research Ethics Committee. Institutional body to which data requests may be sent to: Maha Driss. Ethics committee, Immuno Cytopathologic Department, Salah Azaiz Institut, Tunis, Tunisia (mdriss808@gmail.com). The datasets presented in this study can be found in online repositories. The names of the repositories and accession numbers can be found below ovarian carcinoma from the TCGA database (https://www.cancer.gov/ccg/research/genomesequencing/tcga).

## Ethics statement

The studies involving humans were approved by Ethics committee, immuno cytopathologic department, salah Azaiez Institut, Tunis, Tunisia. The studies were conducted in accordance with the local legislation and institutional requirements. The human samples used in this study were acquired from primarily isolated as part of your previous study for which ethical approval was obtained. Written informed consent for participation was not required from the participants or the participants’ legal guardians/next of kin in accordance with the national legislation and institutional requirements.

## Author contributions

AJ: Writing – original draft, Software, Methodology, Investigation, Formal Analysis, Data curation, Conceptualization. RR: Writing – review & editing, Formal Analysis, Methodology. MaM: Writing – review & editing, Supervision, Conceptualization. GS: Writing – review & editing, Supervision, Methodology, Conceptualization. FG: Writing – review & editing, Methodology, Formal Analysis. LC: Writing – review & editing, Validation, Formal Analysis, Data curation. AM: Writing – review & editing, Resources, Funding acquisition. MoM: Writing – review & editing, Visualization, Validation. KM: Writing – review & editing, Visualization, Validation, Resources, Project administration, Funding acquisition. RD: Writing – review & editing, Visualization, Validation, Supervision, Methodology, Investigation, Conceptualization.
