# Supplementary material for: VISTA/CTLA4/PD1 coexpression on tumor cells confers a favorable immune microenvironment and better prognosis in high-grade serous ovarian carcinoma
Source: Front Oncol. 2024 Apr 3;14:1352053. doi: 10.3389/fonc.2024.1352053 (PMC11022690; doi:10.3389/fonc.2024.1352053)
Supplement: Supplementary file 1 [file DataSheet_1.docx]

**Table S1:** Patient clinicopathological characteristics

|  | N (%) |
| --- | --- |
| Age (year) median  <60  >=60 | 55 (21- 85)  92 (68.7%)  42 (31.3%)  1 NA |
| Stage  I -II  III - IV | 30 (22.2%)  105 (77.8%) |
| Lymph node involvement  Yes  No | 43 (39.1%)  67 (60.9%)  25NA |
| Debulking  Complete  Incomplete | 32 (34.4%)  61 (65.6%)  24 NA |
| Chemotherapy neoadjuvant  Yes  No | 27 (28.4%)  68(71.6%)  40 NA |
| Response to Chemotherapy  sensitive  resistant | 74 (85.1%)  13 (14.9%)  48 NA |
| Distant metastasis  Yes  No | 12 (8.9%)  123 (91.1%) |
| Recidive  Yes  No | 53 (42.7%)  71 (57.3%)  11NA |
| Death  Yes  No | 49 (57%)  37(43%)  49 NA |
| OS Mean (months) | 21 (1 -189) |
| PFS Mean (months) | 16 (1- 85) |
| 2 year OS rate | 51.85% |
| 2 year PFS rate | 35,55% |

NA: not available, OS: overall survival, PFS: progression- free survival

**Figure S1:** The gene encoding VISTA was positively associated with genes encoding (**A):** CTLA4 (*p*<0.01, R=0.21), **(B**): PD1 (*p*<0.01, R=0.26), **(C):** PDL1 (*p*<0.01, R=0.17), (**D)**: CD8 (*p*<0.01, R=0.26), and (**E):** FOXP3 (*p*<0.01, R=0.26) but the correlations were weak (R<0.4).

**Figure S2**: Kaplan- Meier OS curves in HGSOC according double immune checkpoints expressions. **(A):** VISTA/PDL1, **(B):** CTLA4/PD1 and **(C):** CTLA4/PDL1.
