# Supplementary figures and images for: VISTA/CTLA4/PD1 coexpression on tumor cells confers a favorable immune microenvironment and better prognosis in high-grade serous ovarian carcinoma
Source: Front Oncol. 2024 Apr 3;14:1352053. doi: 10.3389/fonc.2024.1352053 (PMC11022690; doi:10.3389/fonc.2024.1352053)

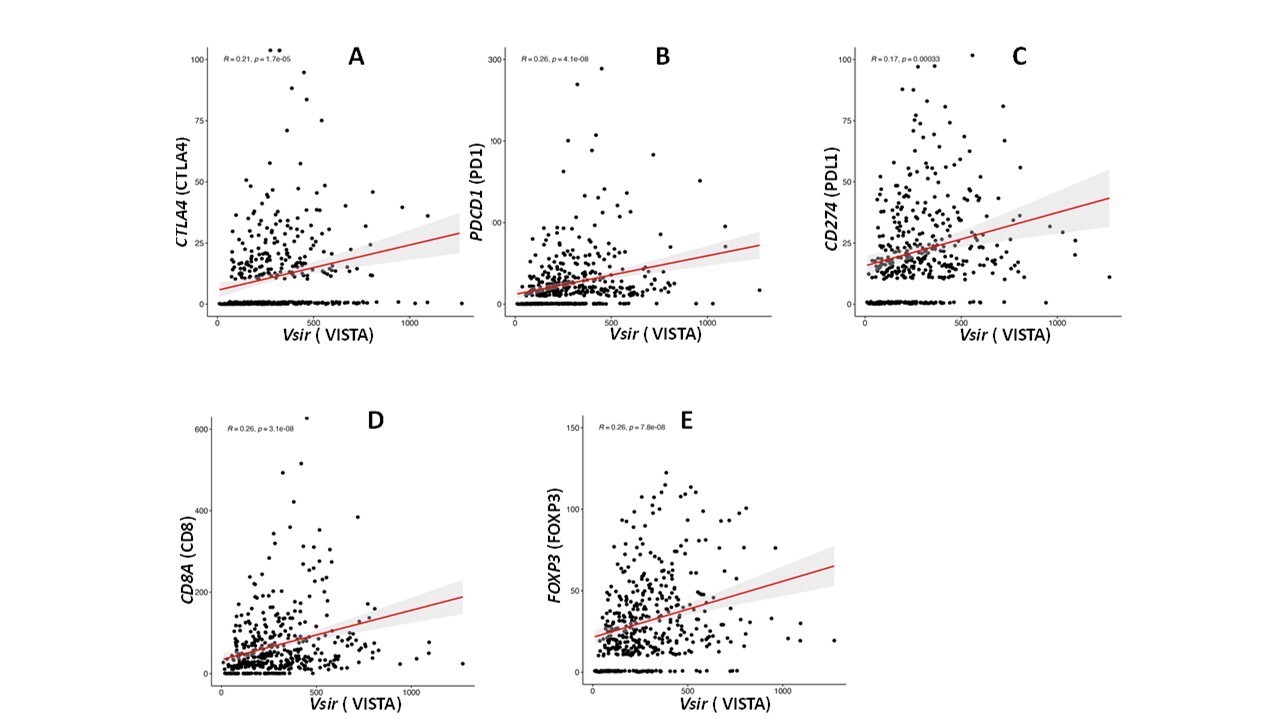


Figure S1


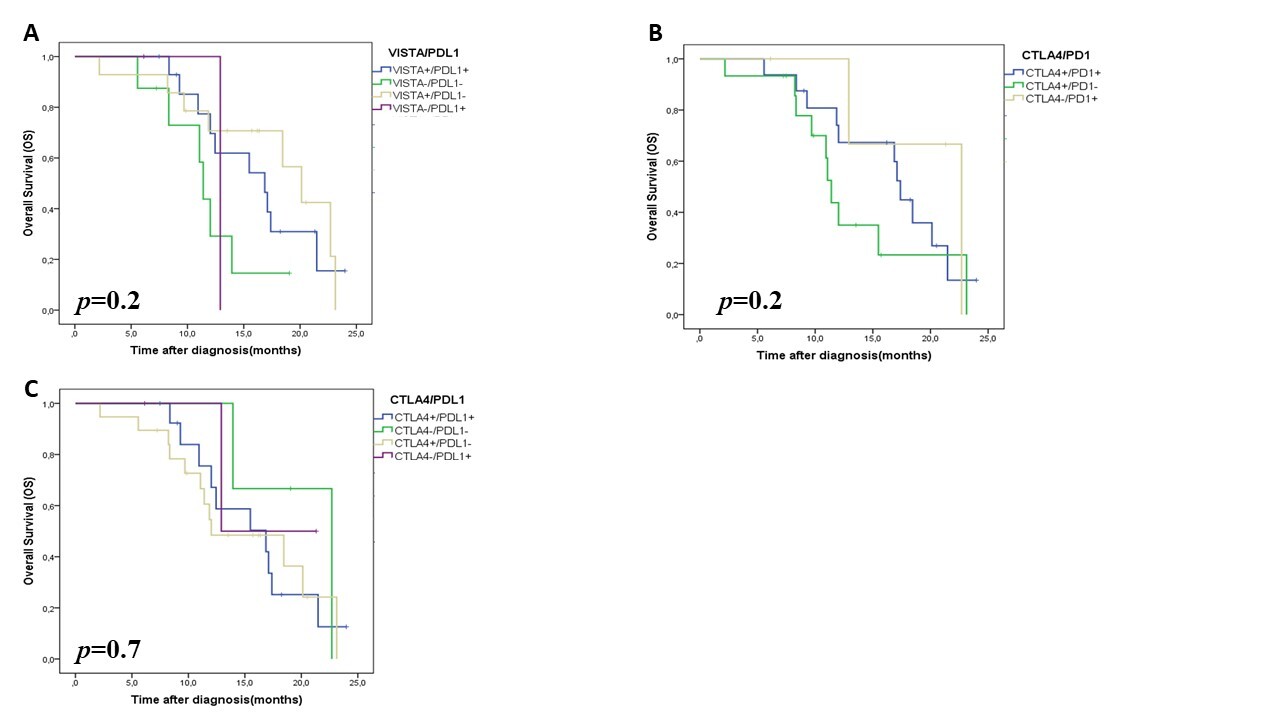


Figure S2

Supplement: Supplementary file 3 [file DataSheet_3.docx]
